# Impact of a High-Fat or High-Fiber Diet on Intestinal Microbiota and Metabolic Markers in a Pig Model

**DOI:** 10.3390/nu8050317

**Published:** 2016-05-23

**Authors:** Sonja N. Heinritz, Eva Weiss, Meike Eklund, Tobias Aumiller, Charlotte M.E. Heyer, Sabine Messner, Andreas Rings, Sandrine Louis, Stephan C. Bischoff, Rainer Mosenthin

**Affiliations:** 1 Institute of Animal Science, University of Hohenheim, Stuttgart 70599, Germany; sonja.heinritz@uni-hohenheim.de (S.N.H.); eva.weiss@uni-hohenheim.de (E.W.); meike.eklund@uni-hohenheim.de (M.E.); tobias.aumiller@delacon.com (T.A.); charlotte.heyer@uni-hohenheim.de (C.M.E.H.); sabinem@uni-hohenheim.de (S.M.); 2 Department of Nutritional Medicine, University of Hohenheim, Stuttgart 70599, Germany; andreas.rings@uni-hohenheim.de (A.R.); sandrine.louis@uni-hohenheim.de (S.L.); bischoff.stephan@uni-hohenheim.de (S.C.B.)

**Keywords:** diet, health, microbiota, pig model

## Abstract

To further elaborate interactions between nutrition, gut microbiota and host health, an animal model to simulate changes in microbial composition and activity due to dietary changes similar to those in humans is needed. Therefore, the impact of two different diets on cecal and colonic microbial gene copies and metabolic activity, organ development and biochemical parameters in blood serum was investigated using a pig model. Four pigs were either fed a low-fat/high-fiber (LF), or a high-fat/low-fiber (HF) diet for seven weeks, with both diets being isocaloric. A hypotrophic effect of the HF diet on digestive organs could be observed compared to the LF diet (*p* < 0.05). Higher gene copy numbers of *Bacteroides* (*p* < 0.05) and *Enterobacteriaceae* (*p* < 0.001) were present in intestinal contents of HF pigs, bifidobacteria were more abundant in LF pigs (*p* < 0.05). Concentrations of acetate and butyrate were higher in LF pigs (*p* < 0.05). Glucose was higher in HF pigs, while glutamic pyruvic transaminase (GPT) showed higher concentrations upon feeding the LF diet (*p* < 0.001). However, C-reactive protein (CRP) decreased with time in LF pigs (*p* < 0.05). In part, these findings correspond to those in humans, and are in support of the concept of using the pig as human model.

## 1. Introduction

Growing attention has been paid to the role of diet, particularly macronutrients, affecting composition and metabolic activity of the human gut microbiota, thereby possibly affecting health, as recently reviewed, for example, by Conlon and Bird [1]. The prevalence of harmful and pathogenic gut microbes has been associated with intestinal disease such as obesity or gastric cancer [2]. Dietary means have proven to be efficient in preserving a healthy gut microbiota population [1], both in terms of short- and long-term effects [3].

Several bacteria such as species of the *Lactobacillus* or *Bifidobacterium* genera [4], or *Faecalibacterium prausnitzii* [5] have shown beneficial effects on the health of humans and animals, and are recognized as biomarkers of intestinal health. On the other hand, the *Enterobacteriaceae* family for example is rather considered to be detrimental for both the human and animal host because of their pathogenic members (e.g., enterotoxic *Escherichia coli* and *Shigella*) [6,7]. For example, activation of the innate immune response has been associated with the presence of lipopolysaccharides (LPS, plasma endotoxins) located on the outer membrane of these gram-negative bacteria [8,9]. Considering this, Myles *et al.* [10] could demonstrate that dietary fat consumption may lead to an increased colonic permeability to gut microbial products such as LPS, which, in turn, may cause immune dysregulation with colonic and systemic inflammation. The role of dietary fat in metabolic endotoxemia is a central issue, which may partly explain the high rate of chronic diseases in Western countries [11,12] as Western-style diets are usually composed of food ingredients rich in fat content [13]. On the other hand, Western-style diets are commonly very poor in dietary fiber content [13]. Dietary fibers are defined as the edible part of plants or analogous carbohydrates that are resistant to digestion and absorption in the human small intestine with complete or partial fermentation in the large intestine. They include polysaccharides, oligosaccharides, lignin and associated plant substances [14]. Major end-products of dietary fiber fermentation are volatile fatty acids including acetate, propionate and butyrate, which reduce the colonic pH, thereby preventing the growth and activity of a variety of pathogenic bacteria [1]. In particular, butyrate serves as main source of metabolic energy for the colonocytes, is supportive in maintaining mucosal integrity, controls intestinal inflammation and endorses genomic stability [15]. 

Though, assessing the impact of diet on the gut microbiota composition, metabolic activity and related processes remains difficult due to restrictions in using human models and the obvious physiological differences between rodents and humans [16]. On the other hand, primate models have stringent ethical restrictions for experimentation compared with mice or pigs [17]. Therefore, the pig as human-sized, omnivorous animal with analogous digestive processes [4] was used as model in the present study. Two different diets, referred to as low-fat/high-fiber (LF), or high-fat/low-fiber (HF), were used to assess their effect on gut microbiota composition and production of microbial metabolites. In addition their effect on the development of digestive organs, and on different biochemical parameters in pigs’ blood serum was determined. This comparative study using LF and HF diets represents an alternative approach to previous studies where, e.g., standard pig diets high in fat, or genetically obese (mini-) pigs were used [18,19,20]. The results of this study are expected to provide more evidence, if and under which conditions the pig can serve as human model to assess the effect of dietary modulations on gut microbiota, biochemical markers, and development of digestive organs.

## 2. Materials and Methods

### 2.1. Ethical Approval

The research protocol was approved by the German Ethical Commission for Animal Welfare (V302/12 TE). All dietary treatments were in accordance with the guidelines issued by the German regulation for care and treatments of animals.

### 2.2. Animals and Housing

Eight castrated male pigs (German Landrace × Piétrain) averaging 3 months in age with an initial body weight (BW) of 27.7 kg ± 1.9 kg were obtained randomly from the Research Station of the University of Hohenheim. Before the start of the experiment, the pigs were acclimated for 10 days to their local environment, *i.e.*, a temperature controlled room (18–20 °C) equipped with infrared heating lamps. Pigs were housed individually in metabolic crates (1.5 m × 1.0 m) permitting visual and olfactory contact between animals, which had free access to water by low pressure drinking nipples. Once daily, groups of 4 pigs each were allowed for about 3 h to move around in an indoor paddock to maintain social contact.

### 2.3. Study Design

In total, 8 pigs were equally and randomly allotted to 2 treatment groups. The first treatment received a HF diet, and the second one was fed a LF diet. Both diets were formulated to meet or to exceed the National Research Council [21] nutrient recommendations for pigs from 25 to 50 kg of BW. The LF diet used in the present study contained 216.8 g neutral detergent fiber (NDF)/kg dry matter (DM), which is about 45% above NDF levels used in standard diets for grower pigs [22]. On the other hand, standard diets usually contain about 30 g·fat/kg·DM [23], whereas the fat content of the HF diet was substantially higher amounting to 249 g·fat/kg·DM. Ingredient composition and nutrient contents of the diets are shown in Table 1. The whole study lasted 7 weeks. Before the start of the experiment, *i.e.*, during the acclimation period of 10 days, pigs received a commercial standard diet based on wheat and barley containing 169.6 g crude protein/kg·DM and 13.23 MJ·ME/kg·DM. Thereafter, pigs were fed the experimental diets for a total of 7 weeks. Animals’ BW was recorded weekly to adjust their daily feed allowances to the assigned feeding level. Daily feed allowance was 3.5% and 4.9% (as fed) of pigs’ average BW for the HF and LF treatment, respectively, to account for differences in gross energy (GE) content between experimental diets (Table 1). As a result, daily calorie intake was the same for all pigs. Pigs were fed 2 equal meals in mash form twice daily (at 0730 and 1530). Blood samples were taken 3 times during the experiment (after 4 and 6 weeks after the start of the experiment, and one day before slaughtering) via the internal jugular vein before the morning feeding. At the end of the experiment, all pigs were taken to a slaughterhouse (butchery Egerhof, Eningen, Germany), and they were processed according to a routine slaughterhouse procedure. After slaughtering, pigs’ empty carcass weight and intestine weights, both full and empty, were measured. Backfat thickness (mean of withers, back midst, loin) was measured as well. Cecal and colonic digesta samples were collected, and all samples were kept on ice approximately 45 min until transferring them to a freezer to be stored at −80 °C for DNA analysis, or at −20 °C for analysis of microbial metabolites.

### 2.4. Chemical and Physical Analysis

Determination of DM, crude ash, crude protein and NDF of the assay diets was performed according to official standard methods [24]. Content of GE in the diets and the fat components butter, margarine and soy oil was measured by means of a bomb calorimeter (IKA calorimeter, C200, IKA^®^-Werke GmbH & Co. KG, Staufen, Germany).

### 2.5. Blood Analysis

Sera were separated from whole blood by centrifugation at 1000× *g* and 4 °C for 10 min and stored at −80 °C until analysis. The following fasting state parameters were measured in a certified medical laboratory (Laborärzte Sindelfingen, Germany): Cholesterol, high-density lipoprotein (HDL), low-density lipoprotein (LDL), triglycerides, glucose i.s., C-reactive protein (CRP), urea, creatinine, Na^+^, K^+^, glutamyl transpeptidase (gamma GT), glutamic pyruvic transaminase (GPT), glutamic oxaloacetic transaminase (GOT) and alkaline phosphatase (AP).

### 2.6. DNA Extraction

Genomic DNA of cecal and colonic samples of each pig (*n* = 4 per treatment) was extracted as described recently by Weiss *et al.* [25] using a combination of the protocol according to Yu and Morrison [26] and the QIAamp^®^ DNA Stool Mini Kit (Qiagen, Hilden, Germany). About 250 mg sample were transferred into a 2 mL screw-cap tube containing 50 mg sterile zirconia beads (Ø 0.5 mm). One milliliter of lysis buffer [26] was added and homogenized on a FastPrep Mini-Beadbeater (MP Biomedicals, Heidelberg, Germany) with 4 m·s^−1^ for 40 s, followed by incubation at 70 °C for 15 min. Samples were gently shaken by hand every 5 min. Thereafter, samples were centrifuged at 16,000× g for 5 min, and 600 μL of the supernatant were transferred to a 2 mL microcentrifuge tube. Another 600 μL of fresh lysis buffer were added to the lysis tube, then homogenization, incubation step and centrifugation were repeated, and the supernatant was pooled. InhibitEX tablet (QIAamp DNA Stool mini Kit, Qiagen, Hilden, Germany) was added to the pooled supernatant and afterwards, extraction of genomic DNA of the samples was performed using the QIAamp^®^ DNA Stool mini Kit (Qiagen, Hilden, Germany) according to the manufacturer’s protocol for stool pathogen detection. Quantity and quality of extracted DNA was determined using a ND-UV-Vis Spectrophotometer (NanoDrop Technologies, San Francisco, CA, USA).

### 2.7. Quantitative Real-Time PCR

Quantitative real-time PCR was performed using previously published primer sets (Table 2). All primers were obtained from biomers.net GmbH (Ulm, Germany). The quantification of total bacteria, *Roseburia* spp., *Lactobacillus* spp., *Bifidobacterium* spp., *Clostridium* Cluster XIVab, *Clostridium leptum* subgroup, the *Bacteroides-Prevotella-Porphyromonas* (*Bacteroides* group), *Enterobacteriaceae*, *Faecalibacterium* *prausnitzii*, *Enterococcus* spp. and *Prevotella* genus was performed using the CFX ConnectTM Real-Time System (Bio-Rad Laboratories GmbH, Munich, Germany) associated with the Bio-Rad CFX ManagerTM Software 3.1 (Bio-Rad Laboratories GmbH, Munich, Germany). Polymerase chain reaction amplification was carried out in 20-μL reaction mixture containing 10 μL KAPA SYBR FAST (PEQLAB Biotechnology GmbH, Erlangen, Germany) and 1 μL template DNA. Corresponding amounts of each primer pair (10 pmol/µL) and BioScience-Grade nuclease-free and autoclaved water were added to achieve respective primer concentrations in the reaction mixture (see Table 2). Standard curves for each primer pair were designed as previously described [25] using serial dilutions of the purified and quantified PCR products generated by standard PCR and genomic DNA from pig feces [27]. The PCR products were purified using the MinElute Purification Kit (Qiagen, Hilden, Germany) and checked by agarose gel electrophoresis (20 g·kg^−1^ agarose) to ensure correct product lengths. Quantity of purified PCR amplification products was determined using Qubit^®^ 2.0 Fluorometer (Invitrogen). Amplification conditions were 95 °C for 3 min for initial denaturation, followed by 40 cycles of denaturation at 95 °C for 5 s, primer annealing for 20 s (annealing temperatures: Table 2) and stepwise increase of the temperature from 65 to 95 °C to obtain melting curve data. Every standard was run in triplicate. Quantification was performed in duplicate, and the mean values were calculated. Results were reported as log_10_ 16S rRNA gene copies/g fresh matter (FM).

### 2.8. Analysis of Microbial Metabolites

Short-chain fatty acid (SCFA) concentrations in cecal and colonic digesta were measured by gas chromatography (HP 6890 Plus GCSystem) using 4-methyl-iso-valerianic acid as the internal standard. Samples (*n* = 4 per treatment) were prepared along the principles described by Zijlstra *et al.* [41] for feces. Ammonia concentration was determined with the aid of a gas-sensitive electrode, combined with a digital voltmeter (Mettler-Toledo): 4 × 2.5 g of homogenized samples were diluted (1:7) with distilled water and centrifuged for 20 min (4750× *g*). The supernatant fluids were pooled, diluted (1:10) and 50 mL of the solution mixed with 0.5 mL of 10 M NaOH. The ammonia released was measured as different voltage in mV.

### 2.9. Statistical Analysis

Homogeneity of variances and normal distribution of the data were confirmed by analysis of the residuals, using the UNIVARIATE procedure of the Statistical Analysis System (SAS, SAS Institute, Inc., Cary, NC, USA). Initially, the following linear model for selecting a repeated correlation structure was considered: yijk = µ + βj + δi + (βδ) ij + εijk; where yijk = jth measurement on kth animal in ith treatment, µ = general term (fixed), βj = effect of jth sampling date (fixed), δi = effect of ith treatment (fixed), (βδ) ij = effect of jth sampling date × ith treatment (random), εijk = error associated with yijk (random). The errors εijk of repeated measurements on the same subject (animal within treatment) are assumed to be serially correlated. Different serial correlation structures were fitted by the REML method as implemented in the MIXED procedure of SAS, and the best structure according to the Akaike Information Criterion was selected. The following models were considered for eijk: independent animal effect (compound symmetry), AR (1) and AR (1) + animal effect. Using the selected correlation structure, the data were subjected to a mixed model analysis using the MIXED procedure of SAS. Furthermore, multiple comparisons among sampling dates within treatments were performed using a *t*-test, with degrees of freedom determined by the Kenward–Roger method [42]. This test was performed only when a preliminary simple *F*-test [43] showed differences at *p* ≤ 0.05 (SLICE = treatment option in MIXED). The same test was used for multiple comparisons among treatments within sampling dates (SLICE = sampling date option in MIXED). Significant differences between treatments were represented by different superscript letters using the algorithm for letter-based representation of all pair wise comparisons according to Piepho [44].

## 3. Results

### 3.1. General Observations

The pigs remained healthy throughout the experiment, and readily consumed their feed allowances. The analyzed chemical composition of the assay diets is shown in Table 1. Pigs’ BW increased with time, yet not differing between treatments.

### 3.2. Effect of Diet Composition on Carcass Parameters

Zootechnical data are shown in Table 3. Body weight at slaughtering day was 72.5 kg for the HF treatment and 77.1 kg for the LF treatment, yet not significantly different (*p* = 0.211), and carcass weight was also similar for both treatments (*p* = 0.890). The empty stomach as well as the empty colon showed a tendency to be heavier in the LF treatment (*p* = 0.078 and *p* = 0.053, respectively). Measurements of full stomach and full colon showed higher weights for the LF treatment (*p* = 0.002) and also weight of liver was higher for the LF compared to the HF treatment (*p* = 0.004). Backfat thickness tended to be higher in the HF pigs (*p* = 0.072).

### 3.3. Effect of Diet Composition on Cecal and Colonic Microbiota

The influence of dietary treatments on bacterial gene copy numbers is shown in Table 4. Total bacteria numbers in cecal contents were higher in the HF compared to the LF treatment (*p* = 0.011), yet abundance did not differ in colon samples. Colonic *Clostridium leptum* gene copy numbers tended to be higher (*p* = 0.056) for the pigs of the LF than for the HF treatment, and were significantly higher (*p* = 0.034) in cecal contents. Also, *Bifidobacterium* spp. occurred in higher numbers in the LF treatment, both in cecum and colon samples (*p* = 0.015 and *p* = 0.008, respectively). For the *Bacteroides* group, higher numbers were found in the HF treatment, both in cecal and colonic digesta, (*p* = 0.013 and *p* = 0.042, respectively), along with higher *Enterobacteriaceae* numbers (*p* < 0.001, cecum and colon) and higher abundance of *Prevotella* spp. in colon samples (*p* = 0.041).

### 3.4. Effect of Diet Composition on Microbial Metabolites in Cecum and Colon

The influence of the dietary treatments on contents of microbial metabolites in cecal and colonic digesta is shown in Table 5. Dietary treatment had a significant effect on concentrations of total SCFA, acetate and butyrate (*p* = 0.023, *p* = 0.013 and *p* = 0.003, respectively), with higher colonic concentrations in LF than in HF pigs. Similarly, cecal samples contained higher levels of acetate and butyrate (*p* = 0.045 and *p* = 0.008, respectively). For ammonia, no differences were found between dietary treatments.

### 3.5. Effect of Diet on Biochemical Parameters in Blood Serum

The influence of the dietary treatment on biochemical parameters in blood serum, measured at three points in time during the experiment, is shown in Table 6. The concentrations of GOT and GPT were higher in the LF pigs compared to the HF treatment (*p* < 0.001). Glucose was higher in the HF pigs compared to the LF treatment (*p* < 0.001), while urea concentrations were higher in LF pigs (*p* = 0.001). Na^+^ also showed higher values in the LF treatment (*p* = 0.002), and LDL/HDL ratio was higher in the LF *versus* the HF pigs (*p* = 0.035). Among the HF pigs, there was a time-dependent effect on concentrations of gamma-GT (*p* = 0.010), glucose (*p* = 0.049), Na+ (*p* = 0.008), and also for HDL (*p* = 0.013), LDL (*p* = 0.008) and LDL/HDL ratio (*p* = 0.005), with increasing values for the lipoproteins over time. Among the LF pigs, GPT, urea, creatinine and HDL concentration increased over time (*p* = 0.002, *p* = 0.002, *p* = 0.021 and *p* = 0.046, respectively), while glucose and CRP decreased (*p* = 0.014 and *p* = 0.042, respectively).

## 4. Discussion

The trophic effect of high-fiber diets on gastrointestinal tract development of growing pigs such as higher stomach and colon weights has been reported repeatedly (e.g., [45,46]). Besides bulking effects, the trophic action of fiber has been attributed to its fermentability, resulting in the production of SCFA [47], thereby stimulating epithelial cell proliferation. In rats, for example, colonic infusion of SCFA showed a trophic effect throughout the intestinal tract [47]. Similarly, Scheppach *et al.* [48] found *in vitro* raised cell proliferation in human cecal mucosa following incubation with SCFA. Furthermore, higher liver weight as observed for pigs of the LF treatment has been associated with increased basal metabolic rate of the animal [46]. In addition, the higher crude protein content in the LF diet (244.3 g/kg·DM) compared to the HF diet (210.2 g/kg·DM) results in higher amounts of nitrogenous compounds being processed by the liver, thus causing a hypertrophic effect on the liver [49]. In line with this finding, increased serum urea concentrations in LF pigs with average concentrations at the upper end of reference values [50], especially at the second and third sampling date, point towards an increased metabolic rate in the liver. It has to be mentioned that high protein diets are increasingly recommended as major management strategy for weight control in overweight and obese individuals, particularly if combined with exercise [51,52,53], since they appear to be effective regarding reductions in appetite, body mass, fat mass and retention of lean mass, at least in the short term [54,55]. In the LF pigs, GPT concentrations were rather elevated in comparison to reference values [50], though still within normal range. Generally, this parameter suggests muscle and liver cell injury, and is increased in most cases of non-alcoholic fatty liver disease in humans [56]. Elevated levels of liver GPT have also been reported by Peters and Harper [57] upon feeding protein-rich (over 35% protein) diets to rats, similar to the conditions in the LF treatment.

The observed increase both in serum GPT concentrations and liver weight suggests a rather detrimental effect on the health status of the pigs compared to the HF treatment. On the other hand, concentrations of CRP, an acute-phase marker for systemic inflammation, decreased with time in the LF treatment. This suggests an improved inflammatory status of the liver. Ismail *et al.* [58] observed higher CRP concentrations in obese humans, linking the chronic low-grade inflammation associated with obesity. Higher BW often results from rather unhealthy nutrition such as consumption of the HF diet, which tended to increase backfat thickness of these pigs compared to the LF pigs. Yet, no increase in CRP concentration was observed for the HF pigs, eventually due to the limited length of the experiment, not allowing the development of a real obesity in HF pigs. Furthermore, despite higher glucose values observed in the HF compared to LF pigs, concentrations were within reference values according to Nerbas [50]. Concerning lipoproteins, one might expect rather reduced LDL levels upon feeding the LF diet due to the high fiber content. However, a lowering effect has especially been ascribed to the consumption of soluble fiber fractions (e.g., [59]) while wheat bran, representing the main constituent of the fiber fraction in this diet, mainly consists of insoluble fiber [60].

Dietary effects on gut microbiota composition revealed lower numbers of total bacteria in cecal digesta of the LF pigs. Similarly, Varel *et al.* [61] observed an initial suppression of the porcine gut microbiota in rectal samples upon feeding a high-fiber diet, followed by an adaption process to the diet with a re-establishment of the microbiota. Moreover, numbers of *Enterobacteriaceae* were lower in the LF in comparison to HF pigs. These gram-negative bacteria are known to induce inflammation through the LPS located on the outer membrane [9]. Here, higher colonic SCFA concentrations in LF pigs, possibly accompanied by a subsequent decrease in luminal pH, might have created an inhospitable environment, e.g., for some acid-sensitive bacteria strains of *E. coli*, which would explain the lower abundance of *Enterobacteriaceae* in LF pigs. Similarly, Smith *et al.* [62] found a decrease in the *Enterobacteriaceae* population in association with higher concentrations of total SCFA in the colon of finisher pigs when feeding barley-based diets compared to oat-based diets. Correspondingly, higher numbers of *Enterobacteriaceae* were found in feces of European children consuming a typical Western diet high in animal protein, sugar, starch and fat but low in fiber compared to children from rural Africa living on more vegetarian diets low in fat and animal protein and rich in starch, fiber, and plant polysaccharides [7]. Still, the different environmental conditions as well as their genetic background have to be considered when comparing humans from different continents.

According to the results of the present study, there were higher values of *Bacteroidetes* (*Bacteroides* group, *Prevotella* spp.) in cecum and colon of the HF pigs. Although differences varied between 0.4 and 0.5 log_10_ only, results pointed towards different proliferation of these bacteria as influenced by diet composition. As recently reviewed by Tjalsma *et al.* [63], the genus *Bacteroides* spp., especially enterotoxigenic *Bacteroides fragilis*, is among bacterial groups possibly being associated with a predisposition for the development of colorectal cancer. In *in vitro* studies, pure cultures of *Bacteroides* strains incubated with human feces stimulated the production of fecapentenes, which are assumed to be co-carcinogens or mutagens [64]. Dietary fat stimulates bile flow, which, in turn, promotes the growth of *Bacteroides* species [65]. This might possibly be an explanation for the enhanced proliferation of this species in cecal and colonic samples of the HF compared to the LF pigs in this study. Furthermore, in a study with human subjects by Wu *et al.* [3], the *Bacteroides* enterotype was highly associated with animal protein, several amino acids, and saturated fats, which suggests that meat consumption according to a Western diet is characteristic for this enterotype. Within this regard, Williams *et al.* [66] recently investigated the impact of solubilized wheat arabinoxylans (AX) added to red meat diets fed to pigs. A counteracting potential of AX could be observed, characterized by reduced protein fermentation and lower microbial production of toxic end products such as ammonia in cecum and colon, while, e.g., the *Bacteroides fragilis* group was relatively high in pigs fed the red meat diet devoid of AX. The results of the present study correspond with those of a study with pigs by Yan *et al.* [19], where a high-fat diet resulted in higher proportions of *Bacteroides* in cecal samples compared to a low-fat diet. On the other hand, there were fewer numbers of *Bacteroides* in genetically obese mini-pigs rather than lean pigs [20], thus pointing towards differences in microbiota composition induced by diet effects and genetically induced obesity.

According to Feng *et al.* [67], dietary fat consumption increased proportion of *Prevotella* in the cecum of growing pigs compared to a basal diet. This result is in line with the present study, yet not expected, since the *Prevotella* genus has mainly been associated with fiber degradation in pigs, as for example reported by Wang *et al.* [68] feeding cellulose-supplemented high-fat diets. Similarly, Zhang *et al.* [69] reported enrichment of the microbiota in obese humans with *Prevotellaceae*, a bacterial group known as potential source of LPS. Schwiertz *et al.* [70] observed a higher proportion of *Bacteroidetes* in the microbiota of obese humans. On the other hand, De Filippo *et al.* [7] found a significant proliferation of *Bacteroidetes* and *Prevotella* in the gut microbiota of children from a rural African village and hypothesized that this could be due to high fiber intake, thereby maximizing metabolic energy extraction from ingested plant polysaccharides. Thus, compared to studies with the pig as animal model, human studies might be less consistent due to varying environmental conditions, genetics, differences in diet composition, and restrictions in the design and standardization of experiments with human subjects. In this respect, Pedersen *et al.* [71] assessed changes in the gut microbiota during development of obesity in cloned *vs.* non-cloned pigs. However, the authors did not observe less inter-individual differences in cloned pigs.

With regard to the rather beneficial members of the gut microbiota, *Faecalibacterium prausnitzii* is a prominent butyrate forming bacteria and dominant member of the subgroup *C. leptum*, being predominant in the colonic microbiota of healthy humans [5]. No differences between treatments were observed for *F. prausnitzii* in the present study, although the *C. leptum* group, including several butyrate producing members [72], showed higher values in cecal samples of the LF treatment. This could be in part due to generally higher abundance of *F. prausnitzii* in feces and lower numbers in mucus, with varying implantation along the gastrointestinal tract [73]. 

There were higher numbers of bifidobacteria in cecum and colon of the LF pigs than in the HF animals. Similarly, there is also evidence from human studies that dietary fiber promotes growth of bifidobacteria (e.g., [74]). Enhanced proliferation of bifidobacteria is considered to be beneficial for the host, as these bacteria produce lactate, which lowers the pH and, in turn, may be metabolized by butyrate producers [75]. Apparently, there exists a relationship between lower numbers of bifidobacteria and overweight and obesity both in adults [70] and children [76] compared to normal weight persons. Interestingly, compared to a standard diet, a high-fat diet also reduced cecal *Bifidobacterium* numbers in mice, which negatively correlated with circulating LPS concentrations and thus metabolic endotoxemia [11]. 

The metabolic activity of the gut microbiota is characterized by the production of various microbial metabolites. In the present study, consumption of the LF diet, supplemented with wheat and wheat bran, resulted in increased acetate and butyrate concentrations in cecum and colon. Lower amounts of acetate and butyrate found in the HF pigs correspond with results of Yan *et al.* [19], who measured lower concentrations of acetate, propionate and butyrate in cecal samples of pigs fed a high-fat diet (17.5% swine grease), compared to pigs fed a low-fat diet (5% swine grease). Possibly, this could be ascribed to an inhibitory effect of fat on fermentation activity, as observed in ruminants [77]. Propionate did not significantly differ between diets in the present study, though a numerically higher concentration of propionate was observed in the cecum of HF pigs. Since the genera *Bacteroides* and *Prevotella* are known propionate producers [69] their higher abundance in HF pigs corresponds to this increase in propionate concentrations.

## 5. Conclusions

In conclusion, the use of the pig as human model that can be supplied with dietary ingredients similar to those used in human nutrition appears to be a promising approach. There were significant differences in the microbial composition, the abundance of several important bacterial groups and metabolites between diets. Our data suggest diet as an important factor that shapes the gut microbial community similar to humans, and the results obtained are in support of the concept to establish the pig as a model for the evaluation of diverse forms of nutrition and dietary components. Within this regard, other parameters associated with obesity, such as abundance of macrophages, neutrophils, T cells, B cells and mast cells in adipose tissue could be further assessed in relationship to diet composition and nutritional habits, as diet-induced obesity is also associated, for example, with hyperleptinemia and resistance to leptin, as reviewed by Kanneganti and Dixit [78]. Moreover, research concerning dietary fiber, possibly holding a protective role against the development of colon cancer [79] could benefit from the use of the pig as a model. Finally, future studies should elaborate if pigs can be used as model to assess the effect of dietary modulation on different metabolic processes, e.g., with respect to energy homeostasis, or factors inducing immune response such as LPS.

## Figures and Tables

**Table 1:** Ingredient composition, nutrient and energy content of the high-fat/low-fiber (HF) and low-fat/high-fiber (LF) diets.

Ingredient, g/kg	HF	LF
Wheat	184.9	477.3
Wheat flour ^a^	200.0	
Wheat bran ^b^	50.0	350.0
Casein ^c^	152.0	120.5
Sunflower margarine ^d^	70.0	
Sweet cream butter ^e^	150.0	
Soy oil	30.0	15.0
Fructose ^f^	50.0	
Dextrose ^g^	50.0	
Cellulose ^h^	30.0	10.0
Vitamin and mineral premix ^i^	17.0	13.4
Potassium chloride	1.4	
Monocalcium phosphate	5.4	
Sodium chloride		0.2
Calcium carbonate	4.3	8.5
Titanium dioxide	5.0	5.0
Analyzed nutrient content		
Dry matter (DM), g/kg	890.3	895.7
Crude protein, g/kg·DM	210.2	244.3
Crude fat, g/kg·DM	248.6	41.3
Neutral detergent fiber, g/kg·DM	66.3	216.8
Gross energy, MJ/kg·DM	23.3	19.2

HF, high-fat/low-fiber; LF, low-fat/high-fiber. ^a^ Siegle GmbH, Ditzingen, Germany; ^b^ BayWa AG, (Nürtingen), Germany; ^c^ Meggle, Wasserburg, Germany; ^d^ REWE Markt, Köln, Germany; ^e^ Milchwerke Schwaben (Weideglück), Neu Ulm, Germany; ^f^ Ferdinand Kreutzer Sabamühle, Nürnberg, Germany; ^g^ Roquette, Frankfurt, Germany; ^h^ Rettenmaier & Soehne, Rosenberg, Germany; from wood; ^i^ Deutsche Vilomix Tierernährung, Neuenkirchen-Vörden, Germany; provided the following quantities of minerals and vitamins per kg HF diet: 4.3 g calcium, 0.9 g phosphor, 0.9 g sodium, 0.2 g magnesium, 6800 I.E. vitamin A, 1020 I.E. vitamin D3, 42.5 mg vitamin E, 0.85 mg vitamin B1, 2.6 mg vitamin B2, 2.1 mg vitamin B6, 17 mcg vitamin B12, 1.7 mg vitamin K3(MNB), 10.6 mg niacin, 6.4 mg Ca-pantothenate, 0.4 mg folacin, 127.5 mg choline chloride, 68.0 mg iron, 8.5 mg copper, 45.4 mg manganese, 56.8 mg zinc-oxide, 1.1 mg iodine, 0.2 mg selenium, 0.1 mg cobalt. For the LF diet: 3.3 g calcium, 0.7 g phosphor, 0.7 g sodium, 0.1 g magnesium, 5360 I.E. vitamin A, 804 I.E. vitamin D3, 33.5 mg vitamin E, 0.67 mg vitamin B1, 2.1 mg vitamin B2, 1.7 mg vitamin B6, 13 mcg vitamin B12, 1.3 mg vitamin K3 (MNB), 8.4 mg niacin, 5.0 mg Ca-pantothenate, 0.3 mg folacin, 100.5 mg choline chloride, 53.6 mg iron, 6.7 mg copper, 35.8 mg manganese, 44.8 mg zinc-oxide, 0.9 mg iodine, 0.2 mg selenium, 0.1 mg cobalt.

**Table 2:** Oligonucleotide primers used for real-time PCR.

Targeted Bacterial Group (Amplicon Size)	Item	Sequence (5′ → 3′)	Annealing Temperature (°C)	Primer Concentration (nM)	qPCR Efficiency (%)	Coefficient of Determination *R*^2^	Reference
Total bacteria (147 bp)	F	GTGSTGCAYGGYYGTCGTCA	52	600	82.6	0.981	Maeda *et al.* [28] ^a^
	R	ACGTCRTCCMCNCCTTCCTC					
*Roseburia* spp. (353 bp)	F	AGGCGGTACGGCAAGTCT	59	400	97.7	0.994	Veiga *et al.* [29]
	R	AGTTTYATTCTTGCGAACG					Rinttilä *et al.* [30]
*Bacteroides-Prevotella-Porphyromonas* (140 bp)	F	GGTGTCGGCTTAAGTGCCAT	59	600	97.2	0.992	Rinttilä *et al.* [30]
	R	CGGAYGTAAGGGCCGTGC					
*Lactobacillus* spp. (391 bp)	F	AGAGGTAGTAACTGGCCTTTA	59	200	89.0	0.986	Malinen *et al.* [31]
	R	GCGGAAACCTCCCAACA					
*Enterobacteriaceae* (385 bp)	F	ATGGCTGTCGTCAGCTCGT	59	600	87.3	0.999	Leser *et al.* [32] ^b^
	R	CCTACTTCTTTTGCAACCCACTC					Sghir *et al.* [33] ^b^
*Clostridium leptum* subgroup (239 bp)	F	GCACAAGCAGTGGAGT	63.3	600	91.6	0.997	Matsuki *et al.* [34]
	R	CTTCCTCCGTTTTGTCAA					
*Clostridium* cluster XIVab (150 bp)	F	GAWGAAGTATYTCGGTATGT	57	600	102.7	0.996	Song *et al.* [35]
	R	CTACGCWCCCTTTACAC					
Genus *Prevotella* (121 bp)	F	GGTTCTGAGAGGAAGGTCCCC	59	600	101.8	0.996	Stevenson & Weimer [36]
	R	TCCTGCACGCTACTTGGCTG					
*Bifidobacterium* spp. (126 bp)	F	CGCGTCCGGTGTGAAAG	59	400	107.0	0.994	Xiang *et al.* [37]
	R	CTTCCCGATATCTACACATTCCA					
*Enterococcus* spp. (144 bp)	F	CCCTTATTGTTAGTTGCCATCATT	59	400	84.0	0.966	Rinttilä *et al.* [30]
	R	ACTCGTTGTACTTCCCATTGT					
*Faecalibacterium prausnitzii* (203 bp)	F	GGAGGATTGACC CCTTCAGT	59	600	85.0	0.985	Balamurugan *et al.* [38]
	R	CTGGTCCCGAAGAAACACAT					

bp, base pairs; F, forward; R, reverse; ^a^ modified by Fuller *et al.* [39]; ^b^ modified by Castillo *et al.* [40].

**Table 3:** Zootechnical data.

	HF	LF	SEM	*p*-Values
Weights				
Body (kg)	72.5	77.1	2.34	0.211
Carcass (kg)	57.6	57.2	1.96	0.890
Organ weights				
Empty stomach (g)	565	690	41.6	0.078
Empty colon (g)	1245	1850	177.9	0.053
Empty cecum (g)	85	135	20.4	0.134
Full stomach (g)	660	1385	98.97	0.002
Full colon (g)	1870	3225	177.19	0.002
Liver (g)	1195	1480	43.4	0.004
Backfat thickness (mm)	16.4	10.3	2.34	0.072

HF, high-fat/low-fiber; LF, low-fat/high-fiber; SEM, standard error of the mean. Values represent least squares means.

**Table 4:** Effect of diet on bacterial numbers in cecal and colonic digesta of pigs (log_10_ 16S ribosomal RNA gene copies/g fresh matter.

		Cecum				Colon			
		HF	LF	SEM	*p*-Value	HF	LF	SEM	*p*-Value
	Total bacteria	10.2	9.9	0.07	0.011	10.3	10.1	0.07	0.063
*Firmicutes*	*Roseburia* spp.	8.0	7.8	0.31	0.675	8.5	7.9	0.26	0.175
	*Lactobacillus* spp.	7.8	8.1	0.11	0.152	7.8	8.0	0.13	0.229
	*Clostridium leptum*	7.4	8.3	0.23	0.034	7.8	8.3	0.17	0.056
	*Clostridium* cluster XIVab	8.8	9.1	0.14	0.135	9.2	9.4	0.13	0.365
	*Enterococcus* spp.	6.5	6.5	0.17	0.912	6.5	6.6	0.14	0.900
	*Faecalibacterium prausnitzii*	6.2	6.8	0.46	0.373	6.3	7.1	0.47	0.300
*Bacteroidetes*	*Bacteroides* group	9.3	8.8	0.09	0.013	9.3	8.9	0.09	0.042
	*Prevotella* spp.	10.0	9.6	0.11	0.050	10.0	9.6	0.10	0.041
*Actinobacteria*	*Bifidobacterium* spp.	5.6	7.5	0.40	0.015	5.6	7.9	0.41	0.008
*Proteobacteria*	*Enterobacteriaceae*	8.4	6.3	0.19	<0.001	8.5	6.4	0.21	<0.001

HF, high-fat/low-fiber; LF, low-fat/high-fiber; SEM, standard error of the mean; values represent least squares means.

**Table 5:** Effect of diet on concentrations of short-chain fatty acids (SCFA) and ammonia in cecal and colonic digesta.

	Cecum				Colon			
	HF	LF	SEM	*p*-Values	HF	LF	SEM	*p*-Values
SCFA, mmol/kg of DM								
Acetate	538.9	764.3	62.98	0.045	218.7	422.2	40.85	0.013
Propionate	494.6	330.0	50.14	0.059	217.9	170.9	20.50	0.156
Iso-Butyrate	6.7	6.5	0.90	0.857	8.1	10.8	1.73	0.315
Butyrate	80.4	201.7	22.12	0.008	59.9	117.8	8.71	0.003
Iso-Valeric Acid	8.5	6.9	1.26	0.407	10.7	13.8	2.69	0.449
Valeric Acid	26.8	15.4	4.11	0.098	26.8	15.5	7.12	0.303
Total	1156.0	1324.8	120.97	0.362	542.1	751.0	48.43	0.023
Ammonia, mmol/kg of DM	21.4	13.4	3.77	0.184	15.2	17.2	2.93	0.645

DM, dry matter; HF, high-fat/low-fiber; LF, low-fat/high-fiber; SEM, standard error of the mean; SCFA, short-chain fatty acids; values represent least squares means.

**Table 6:** Effect of diet on biochemical parameters in blood serum.

Parameter		Sampling Date				*p*-Values	
		1	2	3	Pooled SEM	Diet	Week * Diet (HF or LF)
Gamma-Gt (U/I)	HF	33.75 ^w^	38.75 ^x^	34.25 ^w^	2.91	0.731	0.010
	LF	34.75	34.25	33.50			0.705
GOT (U/I)	HF	21.25	20.75 ^a^	21.50 ^a^	1.94	<0.001	0.962
	LF	25.75	28.67 ^b^	29.75 ^b^			0.349
GPT (U/I)	HF	22.75 ^a^	21.00 ^a^	24.75 ^a^	3.67	<0.001	0.176
	LF	56.50 ^b,w^	60.00 ^b,w^	69.00 ^b,x^			0.002
AP (U/I)	HF	243.75	220.50	221.00	15.24	0.117	0.103
	LF	195.25	199.10	182.75			0.387
Glucose i.s. (mg/dL)	HF	105.50 ^a,x^	95.00 ^a,w^	99.25 ^a,w,x^	2.79	<0.001	0.049
	LF	90.75 ^b,x^	82.75 ^b,w,x^	77.75 ^b,w^			0.014
Urea (mg/dL)	HF	20.25 ^a^	22.50 ^a^	21.75 ^a^	2.59	0.001	0.501
	LF	33.50 ^b,w^	42.25 ^b,x^	42.75 ^b,x^			0.002
Creatinine (mg/dL)	HF	1.12	1.18	1.20	0.059	0.363	0.185
	LF	1.01 ^w^	1.09 ^w^	1.17 ^x^			0.021
Na^+^ (mmol/L)	HF	138.50 ^a,w^	138.75 ^w^	142.50 ^x^	0.88	0.002	0.008
	LF	142.25 ^b^	141.00	144.25			0.054
K^+^ (mmol/L)	HF	4.25	4.70	4.73	0.18	1.000	0.142
	LF	4.48	4.85	4.35			0.145
Cholesterol (mg/dL)	HF	76.00	83.00	89.25	4.69	0.710	0.071
	LF	81.50	87.50	85.75			0.336
Triglycerides (mg/dL)	HF	28.50	21.50	26.25	2.86	0.781	0.056
	LF	23.25	27.75	22.50			0.129
HDL (mg/dL)	HF	38.25 ^w^	40.50 ^w^	44.25 ^x^	1.62	0.098	0.013
	LF	35.00 ^w^	36.75 ^w,x^	39.75 ^x^			0.046
LDL (mg/dL)	HF	42.00 ^w^	48.00 ^w,x^	54.25 ^x^	3.11	0.617	0.008
	LF	47.00	51.25	52.00			0.219
LDL/HDL ratio	HF	1.10 ^a,w^	1.22 ^a,w,x^	1.23 ^x^	0.05	0.035	0.005
	LF	1.33 ^b^	1.40 ^b^	1.33			0.061
CRP (mg/L)	HF	1.03	1.13	1.13	0.15	0.623	0.878
	LF	1.45 ^x^	1.00 ^w^	1.00 ^w^			0.042

HF, high-fat/low-fiber; LF, low-fat/high-fiber; SEM, standard error of the mean. Gamma-GT, glutamyl transpeptidase; GOT, glutamic oxaloacetic transaminase; GPT, glutamic pyruvic transaminase; AP, alkaline phosphatase; HDL, high-density lipoprotein; LDL, low-density lipoprotein; CRP, C-reactive protein. Sampling Date 1 and 2: After four and six weeks. Sampling Date 3: One day before slaughtering. With regard to the diet, data not sharing the same letter (a, b) within a column and for one parameter assessed are significantly different (*p* < 0.05); With regard to the experimental weeks, data not sharing the same letter (w, x, y) within a row are significantly different, for HF or LF (*p* < 0.05). Values represent least squares means.

## Abbreviations

The following abbreviations are used in this manuscript:

AP: alkaline phosphatase
AX: arabinoxylan
Bp: base pairs
BW: body weight
CRP: C-reactive protein
DM: dry matter
F: forward
FM: fresh matter
Gamma GT: glutamyl transpeptidase
GE: gross energy
GOT: glutamic oxaloacetic transaminase
GPT: glutamic pyruvic transaminase
HF: high-fat/low-fiber
LDL: low-density lipoprotein
LF: low-fat/high-fiber
LPS: lipopolysaccharide
NDF: neutral detergent fiber
R: reverse
SCFA: short-chain fatty acids
SEM: standard error of the mean
